# Efficacy and safety of anlotinib combined with immune checkpoint inhibitors and platinum-containing chemotherapy for later-line advanced non-small cell lung cancer: a retrospective three-arm real-world study using propensity-score matching

**DOI:** 10.3389/fonc.2024.1446950

**Published:** 2024-11-25

**Authors:** Zeyang Wang, Bingnan Ren, Haotian Yang, Xuejia Qiu, Yin Wu, Chaojun Xue, Yue Zhao, Xiao Li, Ze Yu, Jinyuan Zhang

**Affiliations:** ^1^ Department of Oncology, Hebei General Hospital, Shijiazhuang, China; ^2^ Department of Pharmacy, Hebei General Hospital, Shijiazhuang, China; ^3^ Hebei Key Laboratory Of Clinical Pharmacy, Shijiazhuang, China; ^4^ Beijing Medicinovo Technology Co., Ltd., Beijing, China

**Keywords:** anlotinib, immune checkpoint inhibitors, PD-1/PD-L1, angiogenesis inhibitors, combination therapy

## Abstract

**Objective:**

To assess the efficacy and safety of anlotinib combined with immune checkpoint inhibitors (ICIs) in patients with advanced non-small-cell lung cancer (NSCLC).

**Methods:**

Clinical data on patients with advanced NSCLC were collected from June 2019 to October 2022 at Hebei General Hospital, China. The efficacy and safety of anlotinib combined with ICIs and platinum-containing chemotherapy were retrospectively analyzed. The primary endpoint was progression-free survival (PFS). The secondary endpoint was the disease control rate (DCR) and overall survival (OS). Survival curves were created using the Kaplan–Meier method. The efficacy and adverse reactions were evaluated according to the RECIST 1.1 and CTCAE 5.0 standards.

**Results:**

A total of 54 patients were enrolled in this study after propensity score matching (PSM), including 27 men and 17 women, with a median age of 59. A total of 26 patients received anlotinib + ICIs + platinum-containing chemotherapy (AIC), 15 patients received anlotinib + platinum-containing chemotherapy (AC), and 13 patients received ICIs + platinum-containing chemotherapy (IC). The PFS of the AIC group was 7.76 months (95% CI: 3.71–NC). The DCR was 65.38%. The OS endpoint had not been reached, The AIC combination regimen group had a significantly longer PFS than the IC group (mPFS, 7.76 vs. 2.33 months, p=0.012, HR=0.23, 95% CI: 0.06–0.8). There was no significant difference in the DCR between the two groups (65.38% vs. 53.85%, p=0.326). There was a statistically significant difference in PFS between the AC group and the IC group (mPFS, 9.2 vs. 2.33 months, p=0.02, HR=0.14, 95% CI: 0.03–0.65). There was no significant difference in the DCR between the two groups (40% vs. 53.85%, p=0.445). The common adverse reactions of the combination of anti-angiogenic agents, ICIs, and platinum-containing chemotherapy were anemia (34.62%), allergic reactions (19.23%), thrombocytopenia (11.54%), gastrointestinal reactions (15.38%), and hepatobiliary disorders (11.54%). Most of them were manageable.

**Conclusions:**

Anlotinib combined with immune checkpoint inhibitors and platinum-containing chemotherapy regimens offers a good survival benefit for patients with advanced non-small-cell lung cancer who fail to respond to standard therapy. When both efficacy and safety are considered, a combination of anti-angiogenic agents, ICIs, and platinum-containing chemotherapy can be used as a choice for the treatment of advanced NSCLC.

## 1 Introduction

Globally, NSCLC represents the most common cancer in men and the third most common cancer in women (1). In China, the age-standardized incidence rates of lung cancer for male and female populations are 48.87 and 23.52 per 100,000, respectively (2).

Vascular endothelial growth factor (VEGFR)-associated multi-targeted tyrosine kinase inhibitors (TKIs) and ICIs have achieved commendable success in treating both NSCLC and SCLC. Angiogenesis inhibitors can effectively inhibit tumor proliferation and metastasis. Anlotinib is an orally administered small-molecule kinase inhibitor that blocks the activity of several protein kinases, including those involved in tumor pathogenesis, such as VEGFR, fibroblast growth factor receptor (FGFR), platelet-derived growth factor receptor (PDGFR), and c-kit (3–5). In large randomized placebo-controlled trials, such as the ALTER series (6, 7), anlotinib was associated with a survival benefit in patients with NSCLC who progressed on standard therapies. Therefore, anlotinib represents a potential further line of therapy in this otherwise treatment-refractory population. Anlotinib has been approved as a third-line therapy for NSCLC in China.

ICIs have revolutionized the treatment of NSCLC by harnessing the power of the immune system to target cancer cells (8). These agents block the immune checkpoints that tumors use to evade detection by the immune system, thereby enhancing the immune response against cancer (9). ICIs such as nivolumab and pembrolizumab have been approved for treating advanced NSCLC, demonstrating improved survival outcomes compared to traditional chemotherapy (10). The potential of ICIs in combination with other therapies, including antiangiogenic drugs, is an area of active investigation (11).

Some clinical studies have explored the efficacy of antiangiogenic therapy plus chemotherapy or ICIs in treating NSCLC. Currently, the IMpower150 study (NCT02366143), an open-label phase III randomized controlled trial (RCT), has explored the efficacy of the first-line treatment with chemotherapy plus angiogenesis inhibitors and ICIs in advanced non-squamous NSCLC. The results showed that the combined regimen had favorable clinical effects compared to the non-combined treatment regimen (12). Some scholars have started to study combination therapy with anlotinib and ICIs for advanced solid tumors (13, 14). The combination of anti-angiogenic agents, ICIs, and platinum-containing chemotherapy is effective and well tolerated in the second- or later-line treatment of advanced solid tumors.

In this retrospective study, we conducted a three-arm retrospective real-world analysis of patients receiving anlotinib, ICIs, and platinum-containing chemotherapy who had progressed on more than two lines of therapy at our institution.

## 2 Materials and methods

### 2.1 Study design

This was a retrospective, single-center, real-world study to evaluate the effectiveness and safety of anlotinib, ICIs, and platinum-containing chemotherapy for patients with advanced NSCLC. A total of 67 patients with advanced lung cancer were included between June 2019 and October 2022. The study was conducted following the Declaration of Helsinki and was approved by the ethics committee institutional review board of Hebei General Hospital. Informed consent from patients was exempted from the ethical review.

### 2.2 Patients

All patients were aged 18–80 years, had histopathologically or cytologically confirmed advanced primary NSCLC according to the Guidelines for the Diagnosis and Treatment of Primary Lung Cancer (2022 edition) in China, and received at least two cycles of combined therapy for the study.

The inclusion criteria were as follows (1): age ≥ 18 years (2), primary non-small-cell lung cancer diagnosed by cytology or histology, and (3) hospitalization ≥2 times.

The exclusion criteria were as follows (1): patients who did not receive the combination of anlotinib + ICIs + platinum-containing chemotherapy, anlotinib + platinum-containing chemotherapy, or ICIs + platinum-containing chemotherapy regimens (2); TNM staging for patients in stages I and II; and (3) patients with missing key data (such as organizational credit type).

**Figure 1:**
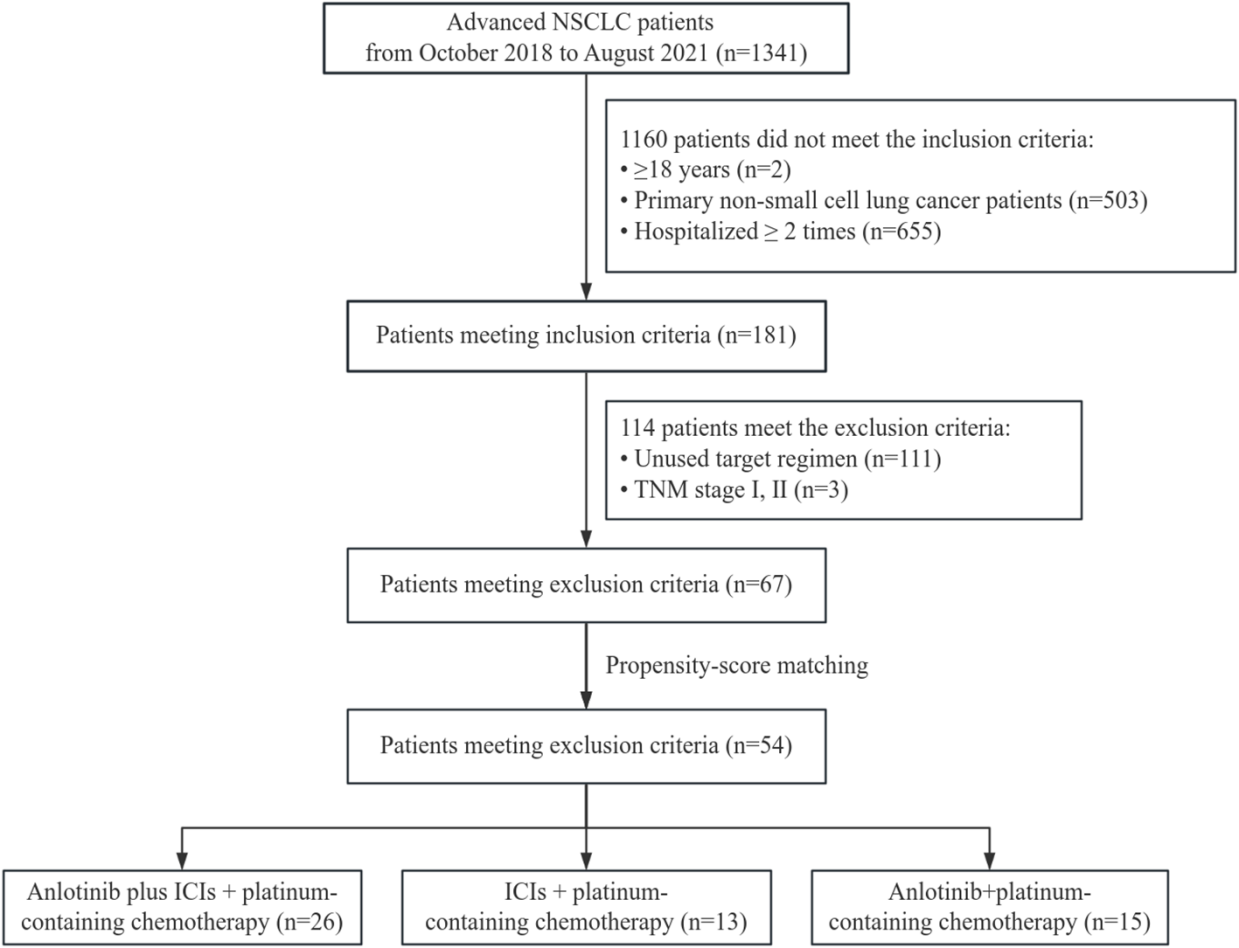
Flow chart of the retrospective study.

Patients were also required to have survival data, adverse events (AEs), and at least one follow-up radiological information (computed tomography). Patients who underwent pregnancy or lactation were excluded from this study. The flowchart of the retrospective study is shown in **Figure 1**.

### 2.3 Procedures and treatment

The study patients were divided into three groups according to the medication plan: AIC, AC, or IC regimen. Anlotinib was given orally once daily with an initial dose of 8–12 mg (day 1–14, every 3 weeks per cycle; Chia-tai Tianqing Pharmaceutical Co., Ltd., Nanjing, China). The ICIs, including Sintilimab (200 mg every 3 weeks; Jiangsu Hengrui Pharmaceutical Co., Ltd., Lianyungang, China), Camrelizumab (200 mg every 3 weeks; Jiangsu Hengrui Pharmaceutical Co., Ltd., Lianyungang, China), Tislelizumab (200 mg every 3 weeks; BeiGene Co., Ltd., Shanghai, China), Nivolumab (3 mg/kg every 3 weeks; Bristol-Myers Squibb), and Pembrolizumab (200 mg every 3 weeks; MSD R&D (China) Co., Ltd., Shanghai, China), were administered via an intravenous drip. The intravenous platinum chemotherapy consisted of 40 mg/m^2^ infusions of Cisplatin (Qilu Medicine Co., Ltd., China) or AUC 4–6 infusions of Carboplatin (Qilu Medicine Co., Ltd., China) for 1 h. Discontinuation, suspension, and dose modification were allowed according to disease progression or AEs.

### 2.4 Treatment evaluation

Information on the patient’s demographic characteristics, laboratory test results, radiological information, survival data, and AEs was collected retrospectively. The tumor response was assessed by the investigator according to the Response Evaluation Criteria in Solid Tumors (RECIST) version 1.1 using computed tomography scans.

The primary endpoint was PFS, defined as the time from the first medication to the occurrence of disease progression or death from any cause. The secondary endpoints included the DCR, OS, and safety. The DCR was defined as the proportion of patients with confirmed complete response, partial response (PR), or stable disease (SD) at the best response. Progressive disease was defined radiographically based on the radiologist’s interpretation. Disease control was defined radiographically as stable disease or partial response, based on the radiologist’s interpretation. The OS was defined as the time from the first medication to the occurrence of death. The Kaplan–Meier method was used to estimate PFS and OS, and Cox proportional hazards modeling was used to evaluate predictors of those outcomes.

Safety was assessed by AEs according to the Common Terminology Criteria for Adverse Events version 5.0 (CTCAE 5.0). If the patient acquired disease progression, serious AEs, or drug toxicity, the drug should be discontinued immediately.

### 2.5 Statistical analysis

To eliminate confounding factors, the control test and control groups were matched by the PSM method. The control variables include gender, age, TNM stage, and histological types.

Quantitative data are statistically described using the number of cases, mean, standard deviation, median, minimum and maximum, and upper and lower quartiles. Categorical indicators are statistically described using the number and percentage of patients in each category. All data are described as median (quartile 2) and quartiles 1 and 3 (Q1–Q3). For multiple-choice categorical indicators, the number and proportion of cases in each category are listed separately.

When describing qualitative or hierarchical indicators, we list the frequency and percentage. For comparisons of unordered categorical indicators, we use the chi-square test or exact probability method (Fisher’s method).

All the statistical tests were performed using two-sided tests, with the test statistics and corresponding p-values given. When using the exact probability method (Fisher’s method), the p-value was directly given. A p-value ≤0.05 was considered statistically significant for the difference tested.

The SPSS software (version 21.0, SPSS Institute. IL., USA) was used for statistical analysis. PFS and OS were calculated by the Kaplan–Meier method and compared using a stratified log-rank test. The analysis of ORR and DCR was based on the best overall response. p<0.05 was considered significant.

## 3 Results

### 3.1 Demographic characteristics

A total of 1,341 patients with NSCLC were admitted to the oncology department during the study period (2019–2022). According to the inclusion and exclusion criteria, 67 patients were included. Among them, 28 patients received anlotinib + ICIs + platinum-containing chemotherapy, 26 patients received anlotinib + platinum-containing chemotherapy, and 13 patients received ICIs + platinum-containing chemotherapy. There were significant imbalances between the three groups in terms of gender and TNM stage, which were recognized as strong risk factors for the outcome and were addressed through PSM. To ensure the balance of the baseline in pairwise comparison and to increase precision, we adopted a consistent matching ratio of 2:1, which was achieved by calculating the difference within each matched set between the patients’ outcome in the intra-group and the mean outcome among the inter-group (15).

After matching according to the ratio of 2:1, 54 samples remained: 26 patients received anlotinib + ICIs + platinum-containing chemotherapy, 15 patients received anlotinib + platinum-containing chemotherapy, and 13 patients received ICIs + platinum-containing chemotherapy. The baseline characteristics of the patients are shown in **Tables 1**, **2**.

**Table 1:** Baseline characteristics of study population before and after PSM with the anlotinib + ICIs + platinum-containing chemotherapy group or the ICIs+ platinum-containing chemotherapy group.

	Baseline comparison before matching				Baseline comparison after matching			
	Anlotinib plus ICIs + platinum- containing chemotherapy(N=28)	ICIs + platinum- containing chemotherapy (N=13)	Statistics	P-value	Anlotinib + ICIs + platinum- containing chemotherapy group (N=26)	ICIs + platinum- containing chemotherapy group (N=13)	Statistics	p-value
Gender								
Male	23 (82.14%)	8 (61.54%)	χ²=1.079	0.299	21 (80.77%)	8 (61.54%)	Fisher	0.253
Female	5 (17.86%)	5 (38.46%)			5 (19.23%)	5 (38.46%)		
Age								
Mean (SD)	60.93 (10.46)	57.00 (13.02)	W=125.5	0.116	60.19 (10.48)	57.00 (13.02)	W=122.0	0.165
Median (Q1–Q3)	62.00 (56.00–65.75)	55.00 (50.00–65.00)			62.00 (56.00–64.75)	55.00 (50.00–65.00)		
TNM stage								
III	11 (39.29%)	6 (46.15%)	χ²=0.173	0.678	11 (42.31%)	6 (46.15%)	Fisher	1.000
IV	17 (60.71%)	7 (53.85%)			15 (57.69%)	7 (53.85%)		
Histological types								
Adenocarcinoma	14 (50.0%)	9 (69.23%)	Fisher	0.302	14 (53.85%)	9 (69.23%)	Fisher	0.371
Squamous carcinoma	13 (46.43%)	3 (23.08%)			11 (42.31%)	3 (23.08%)		
Adenosquamous carcinoma	1 (3.57%)	1 (7.69%)			1 (3.85%)	1 (7.69%)		

Before PSM, gender, age, TNM stage, and histological types were statistically different between the anlotinib + ICIs + platinum-containing chemotherapy group and the ICIs+ platinum-containing chemotherapy group. After PSM, all baseline characteristics were balanced between two groups: gender (proportion of men, 80.77% vs. 61.54%, p = 0.253), age [60.19 (10.48) vs. 57.00 (13.02), p = 0.165], TNM stage (proportion of III, 42.31% vs. 46.15%, p = 1.000), histological types [adenocarcinoma (53.85% vs. 69.23%, p = 0.371)]. After matching, 39 cases were included in the PSM model. All covariates were all well matched, there were no statistical difference (p > 0.05).

**Table 2:** Baseline characteristics of study population before and after PSM with the anlotinib + platinum-containing chemotherapy or the ICIs plus platinum-containing chemotherapy.

	Baseline Comparison before Matching				Baseline Comparison after Matching			
	Anlotinib plus platinum-containing chemotherapy (N=26)	ICIs plus platinum-containing chemotherapy (N=13)	Statistics	P-value	Anlotinib plus platinum-containing chemotherapy (N=15)	ICIs plus platinum-containing chemotherapy (N=13)	Statistics	P-value
Gender								
Male	14 (53.85%)	8 (61.54%)	Fisher	0.740	8 (53.33%)	8 (61.54%)	Fisher	0.718
Female	12 (46.15%)	5 (38.46%)			7 (46.67%)	5 (38.46%)		
Age								
Mean (SD)	61.96 (9.98)	57.00 (13.02)	t=−1.283	0.207	60.33 (10.51)	57.00 (13.02)	t=0.722	0.477
Median (Q1–Q3)	62.00 (55.25–68.25)	55.00 (50.00–65.00)			60.00 (55.00–65.00)	55.00 (50.00–65.00)		
TNM stage								
III	8 (30.77%)	6 (46.15%)	Fisher	0.482	5 (33.33%)	6 (46.15%)	Fisher	0.700
IV	18 (69.23%)	7 (53.85%)			10 (66.67%)	7 (53.85%)		
Histological types								
Adenocarcinoma	18 (69.23%)	9 (69.23%)	Fisher	1.000	10 (66.67%)	9 (69.23%)	Fisher	0.37
Squamous carcinoma	6 (23.08%)	3 (23.08%)			4 (26.67%)	3 (23.08%)		
Adenosquamous carcinoma	2 (7.69%)	1 (7.69%)			1 (6.67%)	1 (7.69%)		

Before PSM, gender, age, TNM stage, and histological types were statistically different between the anlotinib + ICIs + platinum-containing chemotherapy group and the ICIs+ platinum-containing chemotherapy group. After PSM, all baseline characteristics were balanced between two groups: gender (proportion of men, 53.33% vs. 61.54%, p = 0.718), age [60.33 (10.51) vs. 57.00 (13.02), p = 0.477], TNM stage (proportion of III, 33.33% vs. 46.15%, p = 0.700), histological types [adenocarcinoma (66.67% vs. 69.23%, p = 0.370)]. After matching, 28 cases were included in the PSM model. All covariates were all well matched; there were no statistical difference (p > 0.05).

### 3.2 Analysis of the efficacy of different treatment regimens

#### 3.2.1 AIC group vs. IC group

The Wilcoxon rank sum test was used to determine if there is a significant difference in age between the AIC group and the IC group. There was no statistically significant difference in age between the two groups. Fisher’s exact probability method was used to compare gender, TNM stage, and histological types between the AIC group and the IC group. There were no statistically significant differences in gender, TNM stage, or histological types between the two groups (**Table 1**).

The Wilcoxon rank sum test was used to determine if there is a significant difference in PFS between the AIC group and the IC group. There was a statistically significant difference in PFS between the two groups, and the median (Q1–Q3) PFS in the AIC chemotherapy group was 3.68 (2.38–7.65), >1.25 (0.99–1.97) in the IC group (p=0.001).

**Figure 2:**
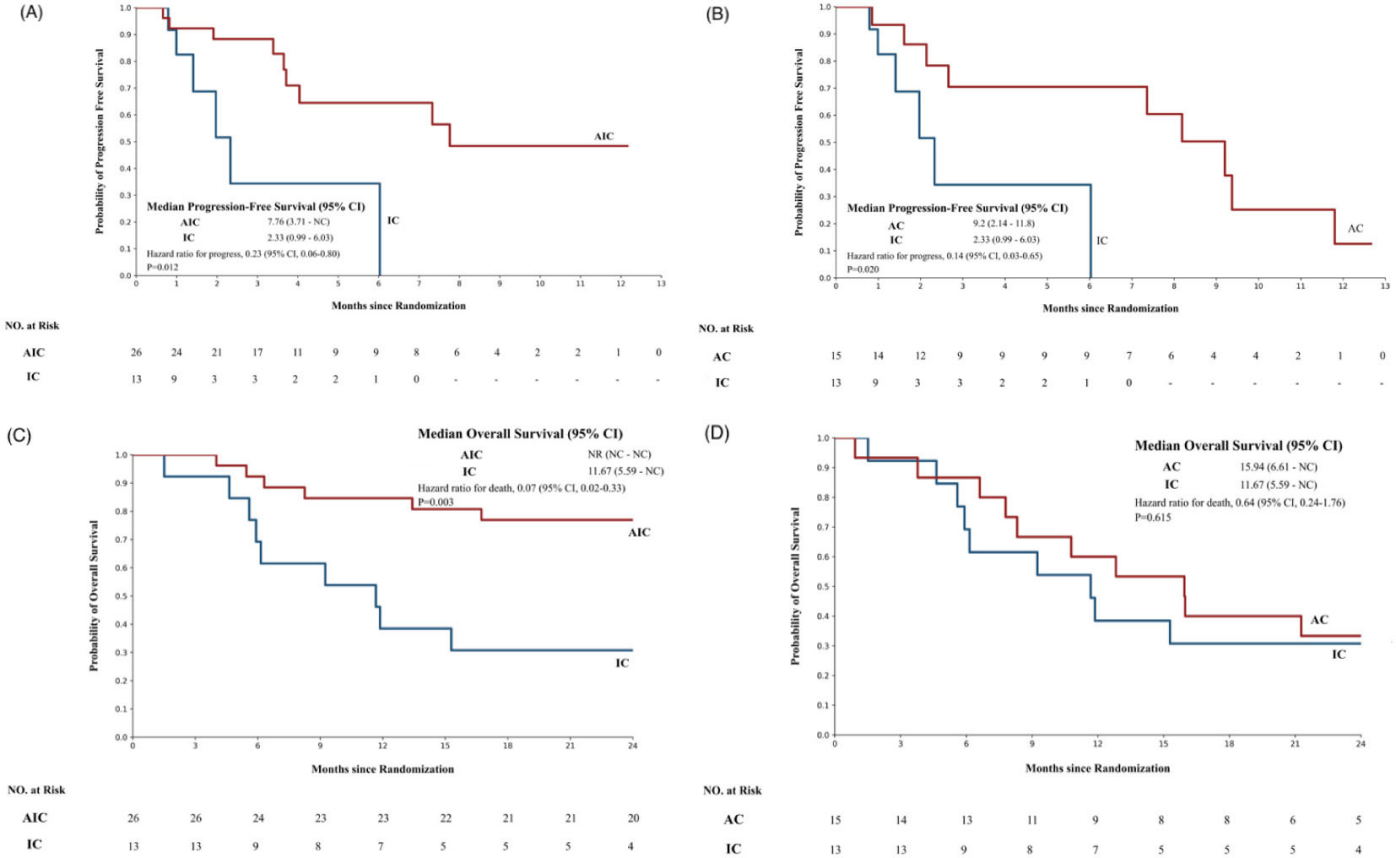
The Kaplan–Meier survival curve of different groups. **(A)** The progression-free survival of patients treated with the anlotinib + ICIs + platinum-containing chemotherapy group or the ICIs+ platinum-containing chemotherapy group. **(B)** The progression free survival of patients treated with the anlotinib + platinum-containing chemotherapy or the ICIs plus platinum-containing chemotherapy. **(C)** The overall survival of patients treated with the anlotinib +ICIs+ platinum-containing chemotherapy group or the ICIs+ platinum-containing chemotherapy group. **(D)** The overall survival of patients treated with the anlotinib + platinum-containing chemotherapy or the ICIs + platinum-containing chemotherapy. AIC, anlotinib + ICIs + platinum-containing chemotherapy; IC, ICIs + platinum-containing chemotherapy; AC, anlotinib + platinum-containing chemotherapy. In this study, the patients were divided into three groups according to the medication situation and were compared by pairwise control. The analysis was conducted after PSM of the 2:1 matching ratio(N = 54).

There was a statistically significant difference in PFS between the two groups, and the median PFS of the AIC group was 7.76 months (3.71–NC) and >2.33 months (0.99–6.03) in the IC group (p=0.012). The hazard of progression was 0.23 (95% CI, 0.06–0.8). Furthermore, there was a statistically significant difference in OS between the two groups. The OS endpoint of the AIC group had not been reached, and the median OS of the IC group was 11.67 months (5.59–NC). The Kaplan–Meier survival curve is shown in **Figure 2**.

In this study, the AIC group shows good anti-tumor activity with a favorable response rate and a tolerable toxicity profile in patients with advanced NSCLC, of which 5 (19.25%) achieved PR, 12 (36.15%) achieved SD, and 9 (34.62%) achieved PD. The DCR was 65.38%, which was not obviously different from that of the IC group (53.85%, p=0.326).

#### 3.2.2 AC group vs. IC group

The t-test was used to compare the age difference between the AC group and the IC group, and there was no statistically significant difference in age between the two groups. Fisher’s exact probability method was used to compare gender, TNM stage, and histological types between the AC group and the IC group. There were no statistically significant differences in gender, TNM stage, or histological type between the two groups (**Table 2**).

The Wilcoxon rank sum test was used to compare the differences in PFS between the AC group and the IC group. There was a statistically significant difference in PFS between the two groups, and the median (Q1–Q3) PFS in the AC group was 3.62 (2.12–8.71) months and >1.25 (0.99–1.97) months in the IC group (p=0.001). There was no significant difference in OS between the two groups [median OS (Q1–Q3): 15.94 (8.05–29.54) vs. 11.67 (5.92–34.81), p=0.548].

There was a statistically significant difference in PFS between the AC group and the IC group, and the median PFS of the AC group was 9.2 months (2.14–11.8), >2.33 months (0.99–6.03) in the IC group (p=0.02). The hazard of progression was 0.14 (95% CI, 0.03–0.65). The Kaplan–Meier survival curve is shown in **Figure 2**.

The AC group showed similar anti-tumor activity to the IC group, of which one (6.67%) achieved PR, five (33.33%) achieved SD, and nine (60%) achieved PD. The DCR was 40%, which was not different from that of the IC group (53.85%, p=0.445).

## 4 Safety of different treatment regimens

In this study, we also evaluated the safety of anlotinib for the treatment of patients. AEs observed in these groups are summarized. The most common AEs are shown in **Table 3**. Among the three groups, myelosuppression was the most common adverse event. The AIC group had the highest incidence rate: 11 (42.31%) patients had neutropenia, 9 (34.62%) patients had anemia, and 3 (11.54%) patients had thrombocytopenia. Other adverse reactions with an incidence >10% were allergic reactions (19.23%), hypertension (15.38%), other gastrointestinal reactions (15.38%), and hepatobiliary disorders (11.54%). In the AIC group and AC group, four (15.38%) patients and five (33.33%) patients had hypertension, respectively. No adverse reactions of hypertension occurred in the IC group without anlotinib. Patients in the AIC and IC groups with ICIs had hepatobiliary disorders, hypothyroidism, pulmonary infection, and other immune-related AEs. The AC group without ICIs did not experience any of the above AEs.

**Table 3:** Comparison of safety between different groups.

	Anlotinib plus ICIs + plus platinum-containing chemotherapy (N=26)	ICIs plus platinum-containing chemotherapy (N=13)	Anlotinib plus platinum-containing chemotherapy (N=15)
Neutropenia	11 (42.31%)	9 (69.23%)	6 (40.0%)
Anemia	9 (34.62%)	4 (30.77%)	5 (33.33%)
Thrombocytopenia	3 (11.54%)	2 (15.38%)	4 (26.67%)
Hypertension	4 (15.38%)	0 (0)	5 (33.33%)
Gastrointestinal reactions	4 (15.38%)	6 (46.15%)	2 (13.33%)
Allergic reactions	5 (19.23%)	1 (7.69%)	3 (20.0%)
Cough	2 (7.69%)	3 (23.08%)	1 (6.67%)
Hepatobiliary disorders	3 (11.54%)	3 (23.08%)	0 (0)
Vomit	1 (3.85%)	2 (15.38%)	2 (13.33%)
Nausea	1 (3.85%)	2 (15.38%)	2 (13.33%)
Hypothyrea	2 (7.69%)	3 (23.08)	0 (0)
Fatigue	1 (3.85%)	2 (15.38%)	0 (0)
Dyspnea	2 (7.69%)	1 (7.69%)	0 (0)
Localized edema	1 (3.85%)	2 (15.38%)	0 (0)
Rash	0 (0)	1 (7.69%)	2 (13.33%)
Pulmonary infection	1 (3.85%)	2 (15.38%)	0 (0)
Immune-related adverse events	1 (3.85%)	1 (7.69%)	0 (0)
Proteinuria	1 (3.85%)	1 (7.69%)	1 (6.67%)
Fever	0 (0)	1 (7.69%)	0 (0)
Chest distress	1 (3.85%)	0 (0)	0 (0)
Diarrhea	0 (0)	1 (7.69%)	0 (0)

## 5 Discussion

Due to the aggressive nature of non-small-cell lung cancer, patients with advanced disease who have undergone multiple chemotherapy treatments often do not respond well to treatment. Immune checkpoint inhibitors or antiangiogenic monotherapy have had only a limited response. For those patients with better performance status, a more intense combination of therapies is expected to result in a better response and prognosis.

This study is intended to evaluate the efficacy and safety of the combination of anti-angiogenic agents, ICIs, and platinum-containing chemotherapy in advanced NSCLC. We explored this issue through a retrospective analysis of clinical data. In the present study, we conducted a three-arm retrospective real-world analysis of patients taking anlotinib and ICIs and platinum-containing chemotherapy who had progressed on prior lines of therapy at our institution. Our results demonstrated the efficacy of anlotinib + ICIs + platinum-containing chemotherapy, as shown by the DCR of 65.38% with a median PFS of 7.76 months (95% CI, 3.71–NC), and the median OS has not been reached (**Figure 2**). The median PFS in our cohort was superior to that in patients in the earlier real-world cohort (PFS, 6.9 months; DCR, 86.6%) (16). This may be because more patients in our cohort started treatment at earlier TMN stages (stage III, 37.31% vs. 16%) (**Table 1**).

Moreover, compared with the ICI combined with the platinum-containing chemotherapy group, the combination of anti-angiogenic agents, ICIs, and platinum-containing chemotherapy combination regimen group had a significantly longer PFS (mPFS, 7.76 vs. 2.33 months, p=0.012, HR=0.23, 95% CI: 0.06–0.8). The DCR of the anlotinib + ICIs + platinum-containing chemotherapy group was 65.38%, which was not different from that of the ICIs + platinum-containing chemotherapy group (53.85%, p=0.326).

Furthermore, there was a statistically significant difference in PFS between the anlotinib + platinum-containing chemotherapy group and the ICIs + platinum-containing chemotherapy group. The median PFS of the anlotinib + platinum-containing chemotherapy group was 9.2 months (range, 2.14–11.8 months), which was longer than 2.33 months (range, 0.99–6.03 months) in the ICIs+ platinum-containing chemotherapy group (p = 0.02). The hazard of progression was 0.14 (95% CI, 0.03–0.65). Immune checkpoint inhibitors plus chemotherapy have not shown better efficacy for non-small-cell lung cancer (**Figure 2**).

Combination therapy involving anti-angiogenic agents, ICIs, and platinum-containing chemotherapy could be a treatment strategy. All the phase III clinical trials and subsequent updated data analysis support that ICIs plus chemotherapy continued to improve treatment efficacy. The addition of ICIs to standard chemotherapy continued to improve treatment efficacy compared to those in the chemotherapy group. The IMpower150 trial explored the combination of the anti-PD-L1 agent atezolizumab, the angiogenic inhibitor bevacizumab, and chemotherapy (carboplatin and paclitaxel) in the first-line treatment of advanced non-small-cell lung cancer. The addition of atezolizumab to bevacizumab plus chemotherapy significantly improved progression-free survival and overall survival, regardless of PD-L1 expression and EGFR or ALK genetic alteration status (12, 17–20).

For patients with end-stage disease progression who have undergone multiple lines of chemotherapy, ICIs often fail to fully utilize the effects of immunotherapy. Single ICIs are not effective for non-small-cell lung cancer. This finding is similar to that of a previous study (21). The combination therapy of PD-1/PD-L1 has also been shown to improve survival compared to platinum-based chemotherapy in advanced NSCLC, particularly in people with a high tumor mutational burden (TMB). The time to response (TTR) of ICIs is generally >2 months. In previous clinical trials, the average time to respond to immune checkpoint inhibitors was 1.9–3.5 months, depending on factors such as CPS status, disease, and duration of medication (22–24). Further CR cases were detected after 8 months of pembrolizumab treatment, and the results of the KEYNOTE-189 trial also showed that the health status/quality of life began to improve globally at week 21 in the pembrolizumab plus chemotherapy group compared to the placebo plus chemotherapy group (25).

In terms of safety, myelosuppression remains the most common adverse reaction of the AIC regimen. Then, there are allergic reactions, hypertension, and gastrointestinal adverse reactions in that order. The platinum-containing chemotherapy is the foundation of the treatment of non-small-cell lung cancer. The combination of anti-angiogenic agents, ICIs, and platinum-containing chemotherapy can lead to more severe bone marrow suppression, which is consistent with previous research findings (16). In the AIC group and IC group, there were a total of four patients with elevated IL-6 levels. Two of them developed pulmonary infections, one had jaundice, and one had myocarditis. These four patients, in addition to the above adverse reactions, also had elevated CRP levels and decreased blood cell counts, which may be related to immune checkpoint inhibitor-induced cytokine release syndrome (CRS). CRS refers to the phenomenon where ICIs can cause self-targeted immune toxicity by overactivating the immune system, ultimately leading to immune-related adverse reactions (26). IL-6 plays an important role in CRS immunopathogenesis, and the overexpression of IL-6 often signifies CRS (27).

Our cohort experienced a longer PFS than that reported in the ALTER0303 trial (PFS, 5.4 months) (6). This difference might be attributable to the more stringent enrolment criteria and differences in baseline demographics between patients treated with therapies containing ICIs and platinum agents, which are more or less effective for patients with metastatic cancer than chemotherapy treatments not containing ICIs or platinum agents. Our results suggest that the combination of anti-angiogenic agents, ICIs, and platinum-containing chemotherapy may offer better survival outcomes for patients with metastatic cancer compared to the two other therapeutic schedules. However, it is important to note that the optimal treatment strategy for individual patients may vary depending on their specific characteristics and disease status.

This study inevitably has limitations. First, this study was retrospective and involved only one hospital. In addition, this was a small sample study, and although we used propensity scoring to reduce bias, the statistical results were not very convincing, and the optimal patient populations for the combination therapy were not identified. Given the above limitations, our conclusions may require a larger sample size for further confirmation.

## 6 Conclusions

In the real-world setting, the combination of anti-angiogenic agents, ICIs, and platinum-containing chemotherapy is effective and well tolerated in the later-line treatment of advanced NSCLC, and this combination can be used as a treatment choice for advanced NSCLC. The addition of ICIs and anlotinib to the traditional chemotherapy has led to a shift in the approach to treating advanced NSCLC. However, randomized controlled studies are still needed to confirm their efficacy and safety.

## Data Availability

The raw data supporting the conclusions of this article will be made available by the authors, without undue reservation.
